# Forgotten joint score one year after robot-assisted total knee arthroplasty

**DOI:** 10.1007/s11701-026-03340-6

**Published:** 2026-03-23

**Authors:** João Paulo Fernandes Guerreiro, Camila Pinheiro Bortolossi de Souza, Carlos Augusto Ferraresi Sampaio, Giovana Figueiredo Felix Pereira, Paulo Roberto Bignardi, Marcus Vinicius Danieli

**Affiliations:** 1 Faculty of Medicine, Pontifical Catholic University of Paraná, Londrina, PR Brazil; 2 UNIORT.E Orthopaedic Hospital, Londrina, PR Brazil

**Keywords:** Osteoarthritis, Knee, Conventional arthroplasty, Arthroplasty with robotic arm

## Abstract

The objective of this study was to analyze the postoperative results of patients who underwent cTKA compared to those who underwent robot-assisted total knee arthroplasty (raTKA). A retrospective analysis was conducted on patients who underwent primary TKA. Patients were classified into two groups based on prosthesis type. Functional outcomes were assessed using the Forgotten Joint Score (FJS). Statistical analysis included multiple linear regression to identify predictors of joint awareness. A total of 181 patients were analyzed (raTKA group: *n* = 90; cTKA group: *n* = 91). FJS scores were significantly higher in the raTKA group (72.5 [54.1–100]) than in the cTKA group (61.8 [39.6–91.7]; *p* = 0.004). Regression analysis identified the male sex (coef. = 11.50, *p* = 0.005) as a positive predictor of FJS, while cTKA group was associated with lower scores (coef. = − 9.09, *p* = 0.026). Body mass index showed no significant association with FJS (coef. = 0.65, *p* = 0.214). Surgery type and sex influence joint awareness after TKA. Male patients and those undergoing robotic-assisted TKA demonstrated superior functional perception.

## Introduction

Despite significant advances in prosthesis design, surgical techniques, and postoperative rehabilitation protocols over the past two decades, between 10% and 20% of patients remain dissatisfied after total knee arthroplasty using the conventional technique (cTKA) [1]. Widely cited factors related to this dissatisfaction include inaccurate prosthesis positioning and suboptimal restoration of limb alignment [2].

Therefore, robotic technology has been incorporated into knee arthroplasty surgery to help surgeons achieve greater precision in bone cutting, restore mechanical alignment and proper implant positioning, which are important factors in increasing the longevity of the prosthesis and improving functional outcomes [3]. Recent and initial evidence indicate that robotic surgery has provided better clinical outcomes, a lower incidence of complications, and more accurate postoperative limb alignment [1, 3]. The objective of this study was to analyze the postoperative results of patients who underwent cTKA compared to those who underwent robot-assisted total knee arthroplasty (raTKA).

## Materials and methods

This study was approved by the Ethics Committee of the Pontifical Catholic University of Paraná, under opinion 6.903.444. All patients signed informed consent for the use of their data in this study.

The study included patients aged 18 years or older, of both sexes, who underwent primary total knee arthroplasty performed by same knee surgery team. Exclusion criteria included patients who underwent revision arthroplasty, those under 18 years of age, individuals whose medical records were inaccessible, patients who were lost to follow-up during the study period, refused to participate in the study, and, according to the established criteria [2], patients who answered less than 8 of the 12 questions on the assessment questionnaire were excluded.

This was a retrospective cohort study. Between October 2022 and September 2023, all patients who underwent TKA, both by the traditional method and robot-assisted, were selected. The surgical procedures were performed by four orthopedic surgeons, each with more than 10 years of experience in arthroplasty. All procedures cTKA and raTKA followed the same mechanical alignment, targeting a neutral hip-knee-ankle (HKA) angle of 0° ± 3°. No kinematic, restricted-kinematic, or personalized alignment strategies were used. In the raTKA group, bone resections and component positioning were guided by intraoperative robotic planning to achieve the same predetermined mechanical alignment target applied in cTKA. All four surgeons adhered to this standardized protocol.

The data were extracted from electronic medical records and systematically compiled into a standardized database. Patients were classified into two groups according to the type of TKA performed: one group of patients, raTKA, who underwent robotic-assisted total knee arthroplasty (ROSA^®^ Knee System, Zimmer, Warsaw, IN, USA), and another group, cTKA, consisting of those who underwent conventional total knee arthroplasty. All patients received the same prosthesis models (NEXGEN^®^, Zimmer, Warsaw, IN, USA). After classification of the patients, those eligible for participation were contacted by evaluators blinded to the type of surgery performed to agree to the informed consent form, and if so, an adapted questionnaire was administered to assess clinical and functional outcomes. In addition, functional outcomes were assessed using the Forgotten Joint Score (FJS), a validated instrument for hip and knee arthroplasty, culturally adapted to Portuguese [4], which evaluates joint awareness in daily activities and the patient’s perceived knee function and prosthesis integration.

The outcomes analyzed included the results of the FJS-12, pain complaints, return to physical activities, and satisfaction with the surgery. All assessments were performed at least one year after the surgical procedure. As this was a retrospective study, standardized long-leg radiographs were not consistently available across the cohort; therefore, postoperative alignment metrics and preoperative knee phenotype were not collected and could not be used for planning, subgrouping, or comparative analyses.

The Shapiro-Wilk test was used to verify the normality of numerical data. Qualitative data were represented by frequency and analyzed using the t-test or Mann-Whitney test, according to the normality result. Categorical data were represented by mean (SD) or median (IQR) and analyzed using Fisher’s exact test. Multivariate linear regression analysis was also performed to evaluate the factors associated with the outcome. All statistical analyses were performed using STATA v.16 software (Stata Corporation LLC, College Station, USA), with a significance level of 5% for all tests applied.

## Results

**Fig. 1:**
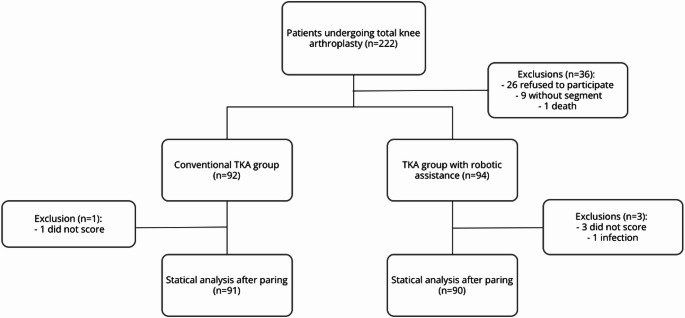
Flowchart of patients recruited for the study

All 222 patients who underwent surgery during the study period were selected, and 186 agreed to participate in the study and were initially included. Of these, 26 refused to answer the questionnaires, 9 were lost to follow-up, and there was 1 death during follow-up. Of the 186 patients recruited, 92 were part of the group that underwent cTKA, and 94 were part of the group that underwent raTKA. Of the 26 patients who refused to participate in the study, 17 were part of the conventional group, and 9 were part of the robot-assisted group. The patient who died belonged to the cTKA group (Fig. 1).

According to the previously established questionnaire validity criteria, five patients were subsequently excluded from the analysis: four from the raTKA group, three due to incomplete responses and one due to postoperative infection, and one from the cTKA group due to an insufficient number of responses.

In the final analysis, therefore, 181 patients were included, totaling 91 patients in the cTKA group and 90 who underwent raTKA. The mean age of the raTKA group was 68.1 ± 8.9, while that of the cTKA group was 68.6 ± 9.0 (Table 1).

**Table 1:** Demographic and clinical characteristics of the patients

Characteristic	raTKA (*n* = 90)	cTKA (*n* = 91)	*p* value
Age, mean ± SD	68.1 ± 8.9	68.6 ± 9.0	0.637
Male sex, n (%)	47 (52.2)	41 (45.1)	0.416
BMI, median (IQR)	29.4 (27.2–32.1)	28.0 (25.0–30.8)	**0.039**

raTKA = robotic-assisted total knee arthroplasty; cTKA = conventional total knee arthroplasty; n = number of patients; BMI = body mass index; IQR = interquartile range

**Fig. 2:**
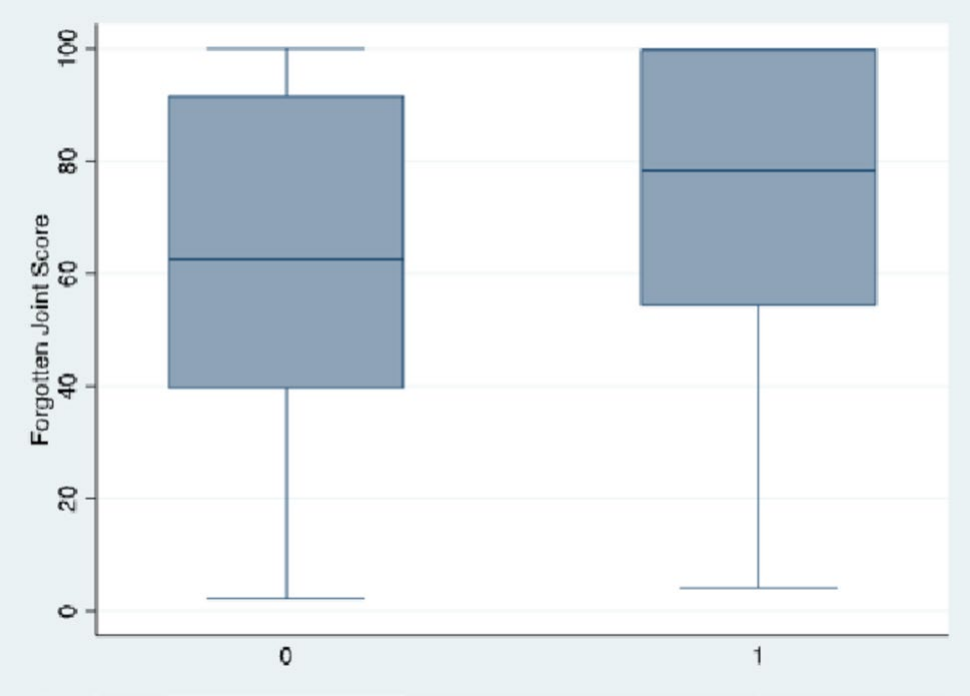
Forgotten Joint Score by type of surgery

As shown in Fig. 2, patient satisfaction with surgery (83.9% vs. 88.5%; *p* = 0.349) did not differ significantly. The FJS showed significantly higher median scores in the raTKA group compared to the cTKA group (72.5 [54.1–100] vs. 61.8 [39.6–91.7]; *p* = 0.004) (Table 2; Fig. 2).

**Table 2:** Patient-reported outcomes

Characteristic	raTKA (*n* = 90)	cTKA (*n* = 91)	*p* value
Satisfaction, n (%)	92 (88.5)	78 (83.9)	0.349
Return to physical activity, n (%)	40 (43.0)	48 (46.1)	0.657
Pain, n (%)	29 (27.8)	34 (37.4)	0.326
FJS, median (IQR)	72.5 (54.1–100)	61.8 (39.6–91.7)	**0.004**

raTKA = robotic-assisted total knee arthroplasty; cTKA = conventional total knee arthroplasty; n = number of patients; FJS = Forgotten Joint Score; IQR = interquartile range

The occurrence of pain was also evaluated between the groups, with 37.4% of patients in the control group reporting this complaint, compared to 27.8% in the robotic group. However, this difference did not reach statistical significance (*p* = 0.326) (Table 2).

The analysis of return to physical activity revealed that, among the patients evaluated, 43.0% in the raTKA group and 46.1% in the cTKA group reported never or rarely remembering the artificial joint while practicing their favorite sport. The difference between the groups, however, was not statistically significant, with a p-value of 0.657, indicating that the return to physical activity and perception of the prosthesis did not vary significantly between the two surgical methods (Table 2).

A multiple linear regression analysis was performed with FJS as the dependent variable. The raTKA group had a regression coefficient of 9.09 (95% CI [1.10–17.1], *p* = 0.026), indicating a significant positive association with FJS compared to the cTKA group. This finding suggests that patients in the raTKA group tend to report higher functional scores. Regarding gender, male patients showed a significant positive association with FJS (coef. = 11.50 95% CI [3.59–19.4], *p* = 0.005), indicating that men tend to report higher FJS scores. On the other hand, no statistically significant association was observed between age and FJS (coef. = 0.15; 95% CI [− 0.29 to 0.59]; *p* = 0.509), nor between BMI and FJS (coef. = 0.65; 95% CI [− 0.38 to 1.68]; *p* = 0.214) (Table 3). Thus, patients in the raTKA group and male participants had superior functional results, reflecting a more favorable perception of their joint function and prosthesis integration.

**Table 3:** Association between the use of robotic-assisted TKA and the functional FJS score

Variable	Coefficient	95% CI	*p* value
Age	0.15	−0.29 to 0.59	0.509
Male sex	11.50	3.59 to 19.40	**0.005**
BMI	0.65	−0.38 to 1.68	0.214
raTKA	9.09	1.10 to 17.10	**0.026**

BMI = body mass index; Coef = coefficient; raTKA = robotic-assisted total knee arthroplasty; CI = confidence interval

## Discussion

Our study demonstrated that patients in the raTKA group and males had better FJS scores, reflecting a more favorable perception of their joint function. Other studies have also demonstrated these differences in functional scores, such as a literature review [5] and a meta-analysis involving 517 patients [1]. However, other studies, including a retrospective observational study and literature reviews [6, 7], failed to show significant differences in functional scores between patients undergoing conventional surgery (cTKA) and those undergoing robot-assisted surgery (raTKA).

According to multiple linear regression, it was possible to observe that the cTKA group had a significant negative regression coefficient with the FJS, compared to the raTKA group. The data indicate that individuals in the cTKA group tend to report worse functional scores. Male participants, on the other hand, showed a significant positive association with FJS, suggesting that men tend to have higher scores. On the other hand, no statistically significant relationship was identified between BMI and FJS. Thus, it can be observed that both undergoing raTKA and being male are factors associated with better functional outcomes, reflecting a more positive perception of joint function and adaptation to the prosthesis, compared to cTKA and females. Another study also reinforces these findings, such as a meta-analysis [8], which found that the raTKA group had higher postoperative FJS than the cTKA group.

Although the raTKA group had a lower incidence of postoperative pain compared to the cTKA group, this difference was not statistically significant. Regarding return to physical activity, the results also showed no statistical differences between the groups. We believe that cohorts with a larger number of patients may demonstrate some statistically relevant differences in these aspects.

Several studies show that robotic surgery is becoming an increasingly prevalent alternative due to its greater surgical precision, as it is possible to provide real-time data and greater standardization of resection, cutting, and bone alignment, thanks to its bone landmark recognition technology [3, 8]. The result would thus be a knee with greater stability, better rotational movement, and no restrictions on important ranges of motion [9]. Despite this, there is still no consensus that the functional results and satisfaction of robotics would be better than conventional methods [3].

In a cohort study conducted by Clement et al. [10], patients undergoing raTKA showed clinically significant improvement in knee pain in the first 12 months postoperatively, with a p-value of 0.029. In addition, these patients were significantly more likely to have their expectations for daytime pain relief met, compared to the group undergoing the conventional technique (*p* = 0.039). These findings suggest that robot-assisted surgery may provide better postoperative pain outcomes and higher patient satisfaction. However, functional outcome scores were not significantly different between techniques [3]. In the present study, the TKA cohort had a considerably lower mean patient satisfaction score, indicating greater satisfaction among patients undergoing the robotic technique.

Our findings are consistent with recent randomized evidence showing clinical benefits of imageless handheld robotic-assisted TKA over conventional TKA at 12 months, including higher functional scores, greater patient satisfaction, and lower pain scores, without an increase in serious adverse events [11]. Notably, that trial also reported a reduced need for ligament releases in the robotic cohort, which may reflect more consistent achievement of the intended mechanical alignment and soft-tissue balance. In the context of return to physical activity and pain, our cohort showed no between-group differences for activity resumption and a non-significant trend toward less pain in raTKA, which parallels the broader literature where early advantages in pain and function may not uniformly translate into large between-group differences at one year [11].

Although our study lacked standardized postoperative radiographs, a randomized trial of imageless handheld robotic-assisted TKA showed significantly fewer > 3° alignment outliers, more accurate achievement of the planned HKA axis, and improved component positioning versus conventional instrumentation [12]. These radiographic advantages offer plausible mechanistic underpinnings for the better patient-reported outcomes observed in our cohort and reported elsewhere.

This study has limitations. First, it is a retrospective cohort study. Since the data were obtained from pre-existing electronic medical records, potentially introducing systematic differences between the study groups, as well as bias arising from patients’ recall during the questionnaires, could also affect the generalization of the results. Subjective outcomes such as the FJS may be influenced by patients’ awareness of having undergone a robotic-assisted procedure, potentially biasing self-reported perceptions of joint awareness and satisfaction. Although assessors were blinded to the surgical technique during follow-up interviews, we cannot exclude expectation bias at the patient level. Despite standardized surgical protocols and multivariable regression adjustment, this retrospective, non-randomized study remains susceptible to residual confounding. Allocation to the cTKA or raTKA groups was determined by routine clinical practice and may have been influenced by unmeasured patient and surgeon-related factors, including baseline functional status, limb alignment, disease severity, surgeon preference, and temporal adoption of robotic technology. Therefore, causal inferences should be interpreted with caution. Another limitation relates to the follow-up time, which does not allow this study to measure the long-term functional and survival outcomes of the prosthesis. In addition the lack of standardized postoperative radiographs precluded objective verification of achieved alignment and component positioning, and the absence of preoperative classification prevented phenotype-based subgroup analyses. These limitations constrain causal inference and the generalizability of our findings. Finally, the sample being composed of patients from a single center, using only one robot and prosthesis model, is also a limitation and restricts the generalization of the findings to other populations.

## Conclusions

In this retrospective cohort study, surgery type and sex influenced joint awareness after TKA. Male patients and those undergoing robotic-assisted TKA demonstrated superior functional perception based on FJS scores.

## Data Availability

No datasets were generated or analysed during the current study.

## Abbreviations

OA: Osteoarthritis
TKA: Total Knee Arthroplasty
cTKA: Conventional Total Knee Arthroplasty
raTKA: TKA with the assistance of a robotic arm
FJS: Forgotten Joint Score
